# Detection of orbital angular momentum using a photonic integrated circuit

**DOI:** 10.1038/srep28262

**Published:** 2016-06-20

**Authors:** Guanghao Rui, Bing Gu, Yiping Cui, Qiwen Zhan

**Affiliations:** 1 Advanced Photonics Center, Southeast University, Nanjing 210096, Jiangsu, China; 2 Electro-Optics Program, University of Dayton, 300 College Park, Dayton, OH 45410, USA

## Abstract

Orbital angular momentum (OAM) state of photons offer an attractive additional degree of freedom that has found a variety of applications. Measurement of OAM state, which is a critical task of these applications, demands photonic integrated devices for improved fidelity, miniaturization, and reconfiguration. Here we report the design of a silicon-integrated OAM receiver that is capable of detecting distinct and variable OAM states. Furthermore, the reconfiguration capability of the detector is achieved by applying voltage to the GeSe film to form gratings with alternate states. The resonant wavelength for arbitrary OAM state is demonstrated to be tunable in a quasi-linear manner through adjusting the duty cycle of the gratings. This work provides a viable approach for the realization of a compact integrated OAM detection device with enhanced functionality that may find important applications in optical communications and information processing with OAM states.

It is well known that light wave can be interpreted quantum mechanically thus can be viewed to carry both spin angular momentum (SAM) and orbital angular momentum (OAM)^1^. Allen *et al.* recognized that light beams with an azimuthal phase dependence of exp(*ilϕ*) carries an OAM that can be many times greater than the spin^2^. Contrary to SAM, which only has two values of ±*ħ* per photon, OAM is equivalent to a value of *lħ* per photon, where *l* can be any integer values. Similar to circularly polarized light, the sign of the OAM indicates its handedness with respect to the beam direction. Since the discovery of optical OAM, light with helical phase front has been used as a useful tool in a variety of applications, ranging from optical spanner, optical tweezers, astronomy, quantum entanglement to microscopy *etc.*^1^,^3^. Furthermore, multiple OAM states could be used as different carriers for multiplexing and transmitting multiple data streams, thereby potentially increasing the system capacity^4^.

In a wide range of applications that involve the use of OAM, it is often crucial to discern different OAM states with high fidelity. Several methods have been demonstrated including using fork diffraction gratings coupled with single mode fiber^5^, cascading Mach-Zehnder interferometers^6^ and transformation optics^7^. However, the experimental setups increase with the highest measurable OAM state. Therefore, the fidelity of the detection is limited by the complexity of the experimental setup and the optical diffraction. Besides, these methods involve the use of bulky elements and complicated procedures that are difficult to be integrated into a compact platform. Photonic integrated circuits (PICs) use light rather than electrons to perform a broad variety of optical functions. Recent developments in nanostructures, metamaterials, and silicon technologies have expanded the range of possible functionalities for these highly integrated optical chips. For example, Mach-Zehnder interferometric and ring-based modulators have been reported^8^,^9^,^10^. In the past few years work has begun on the OAM detection scheme by converting the light with different OAM states into the spatially separated surface plasmon polaritions (SPPs) waves. The unique characteristics of the SPPs in terms of short equivalent wavelength and highly spatial confinement make it possible to develop miniature photonics devices with dimensions much smaller than those are currently available. For example, real-time OAM detection has been demonstrated for metahologram integrated with plasmonic photodiode^11^. This approach enables the realization of a compact and integrated detection device. However, it only works for one specific OAM state. Recently a novel scheme using circular slot optical antenna has been reported to achieve multiple OAM states detection^12^. However, this technique relies on the identification of the SPPs interference pattern, which necessitates a near-field scanning mechanism that leads to slow detection speed. In this work we propose a micro-scale waveguide-based OAM detector, the improved performances of which such as real-time detection, extended detection range and reconfigurability are numerically demonstrated. This silicon-based device has advantages of in low cost and robustness, making it suitable for integrating with each other and other elements to form PICs with specific functionalities.

## Results

### Theory of the OAM detector

**Figure 1:**
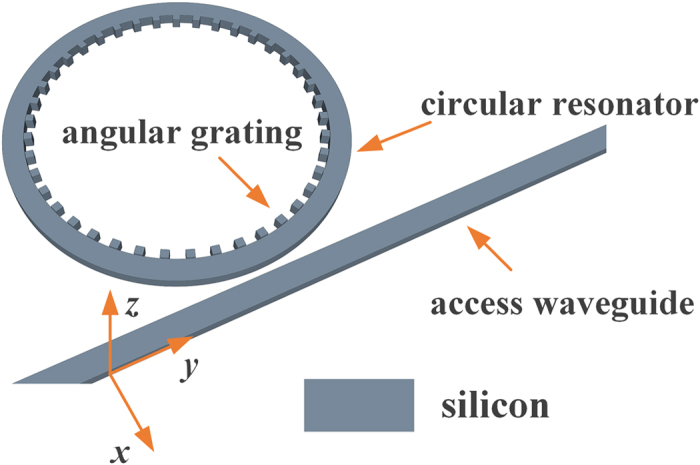
Diagram of the proposed OAM receiver. The circular resonator with angular gratings patterned along the inner wall couples the normally incident azimuthally polarized vortex beam to an access waveguide.

Figure 1 illustrates the diagram of the proposed OAM detector, which consists of circular resonator, angular gratings and access waveguide. The device is made using silicon-on-insulator waveguides, which are 220-nm-thick and 550-nm-width Si on 2 *μ*m of thermal oxide and buried in 2 *μ*m of deposited oxide. Periodic grating elements are patterned on the inner wall of the circular resonator. The width of the gratings is about 1/10 of the grating period (Λ). An access waveguide is placed near the circular resonator with gap size of 150 nm. It is known that whispering gallery resonators support modes with high OAM^13^. However, the low loss of the whispering gallery modes (WGMs) makes it difficult to get light into- or out of- these modes. The periodic variation of the dielectric constant provided by the angular gratings can help to couple the unperturbed eigen-modes of the resonator into other modes. Recently this principle has been applied to the design of compact optical vortex emitter, in which the gratings can scatter the WGMs into free-space propagating optical wave with well-controlled OAM states^14^.

Within the context of antenna theory, the receiver should match to the mode pattern from the emitter to maximize the received signal. This is also true for the presented PIC design for the purpose of detecting OAM states. When the phase and polarization patterns of the illumination match to the corresponding mode patterns that would radiate from the same device as if it were used as an emitter, the received signal will be optimized. In the transmitting mode, various cavity resonances can be excited by the quasi-TE mode in the access waveguide by tuning the injection laser wavelength, leading to the generation of azimuthally polarized vector vortices with quantized and tunable OAM^14^. In turn, the WGM confined in the circular resonator can be excited by the direct illumination in the receiving mode, and then back-coupled to the access waveguide. When the mode matching condition is satisfied, the receiving efficiency of the device can be maximized, which is defined as the ratio of the power coupled to the access waveguide and the incident power, with higher power inside the access waveguide corresponding to higher efficiency.

The characteristics of the proposed device can be described as the coupling between the guided WGM and the illumination mode. According to the coupled mode theory, the topological charge *l* (TC) of the received vortex beam can be expressed as^14^:


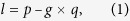


where *p* and *q* are the numbers of the optical periods and grating elements around the circular resonator, respectively, and *g* can be any integer. The value of *p* can be calculated as:


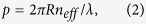


where *R* and *n*_*eff*_ are the inner radius and the effective index of the WGM of the circular resonator, respectively, and *λ* is the wavelength of the illumination. The possible value of *g*, which denotes the diffraction order, is limited by the material system^14^:


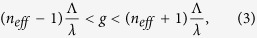


where *n*_*eff*_ is the effective index of the WGM in the circular resonator and *λ* is the wavelength of the illumination. In this design *λ* is around 1550 nm, and *R* is chosen to be 3.9 *μ*m. The grating constant Λ is around 598 nm, and *n*_*eff*_ is simulated to be about 2.6. From equation (3) the value of *g* can only be 1. Therefore equation (1) can be rewritten as:


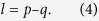


It can be seen that the vortex beam with TC of *l* can be coupled and confined in the *p*-th order WGM. This is a very simple but robust OAM receiver scheme, in which *l* can take any integer value, being determined by the difference between integers *p* and *q*. For example, *p* is calculated to be nearly 41 at the wavelength of 1550 nm. Thus OAM with *l* = 0 can be detected if 41 angular gratings are adopted. By tuning the illumination wavelength to various cavity resonances, variable OAM states can be detected.

### Numerical modeling with three-dimensional finite element method

**Figure 2:**
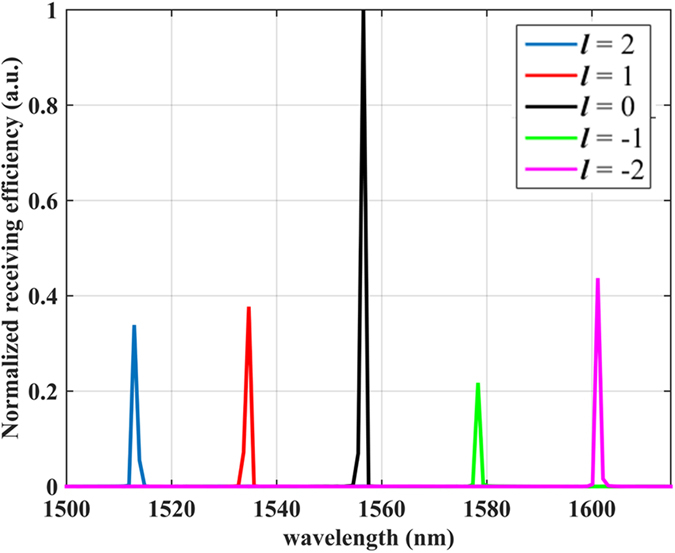
Receiving spectrum of the device shown in Fig. 1.

To study the performance of this OAM receiver and also validate the theoretical predictions, we perform numerical simulation with a three-dimensional finite element method model (COMSOL). An azimuthally polarized vortex beam normally illuminates the device. During the simulation, the OAM receiver is normally illuminated by a vortex beam with transverse size matches the silicon bend waveguide. The receiving efficiency (*M*) is evaluated by comparing the power of the quasi-TE mode in the access waveguide with the power of the illumination. Due to the limited length of the access waveguide considered in the simulation, an *x*−*z* cross section (*R*) of the access waveguide located at *y* = −30 *μ*m is adopted. Therefore, the proportion of power related to TE mode in the access waveguide needs to be considered when evaluating the receiving efficiency:


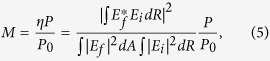


where *η* is the quality of mode matching, *P* is the integrated power over the cross section *R*, *P*_0_ is the incident power, *E*_*f*_ and *E*_*i*_ are the complex electric fields referring to the TE mode and the propagation mode in the access waveguide respectively, and the integration spans the whole cross section. Note that the receiving efficiency can be equivalent to *P*/*P*_0_ if the waveguide is long enough. Figure 2 shows the receiving spectrum of the device for different OAM states from −2 to 2. According to equation (4) each resonance corresponds to a distinctive WGM (*p*) confined in the circular resonator. The receiving efficiencies at various resonances are measured to be 6% to 28%, which vary with wavelength due to the wavelength-dependent coupling between the circular resonator and the access waveguide. Note that the efficiency can be further enhanced by increasing the size of the circular resonator or optimizing the gap distance between the resonator and the access waveguide. In summary, these numerical simulations confirm that the proposed device can be used to detect different OAM states via identifying the resonant wavelengths. Besides, it can also work as a receiver that receiving optical vortex field with high efficiency at the resonant wavelength.

### Effect of the SAM

**Figure 3:**
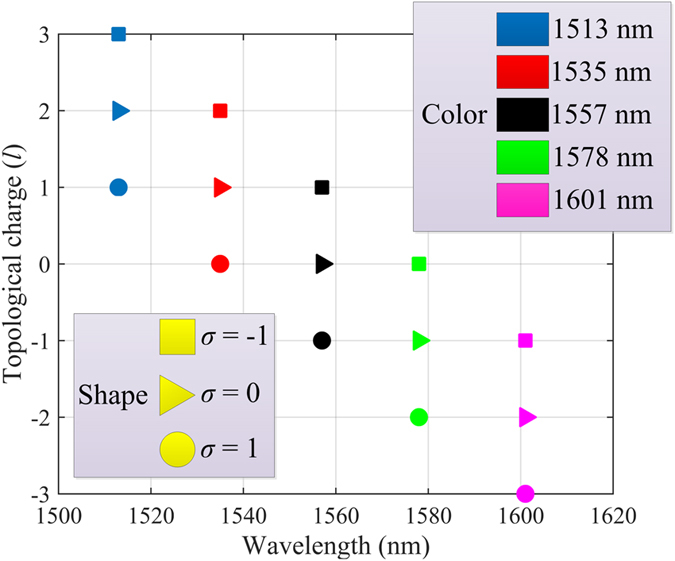
Resonant wavelengths for vortex beam with different SAMs.

With the rapid progress of spin optics, a selection rule associated with angular momentum (AM) attributed to the conservation of total AM in a closed physical system received increasing attention in recent years. Considering a circularly polarized vortex beam normally illuminating the circular resonator, the equation (4) can be rewritten as:


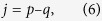


where *j* = *σ* + *l* is the total AM and *σ* denotes the spin state. Since the circular polarization can be decomposed into the combination of radial and azimuthal components along with a geometric phase term, the receiving spectrum of the setup shown in Fig. 2 remains unchanged however the resonant peaks representing *j* instead in this case. As illustrated in Fig. 3, the corresponding OAM of each resonant wavelength strongly depends on the light’s spin. It is also demonstrated the AM conservation law is still valid in this system, and this device is suitable for identifying vortex beam with various polarization states.

### Dual-port detection scheme

**Figure 4:**
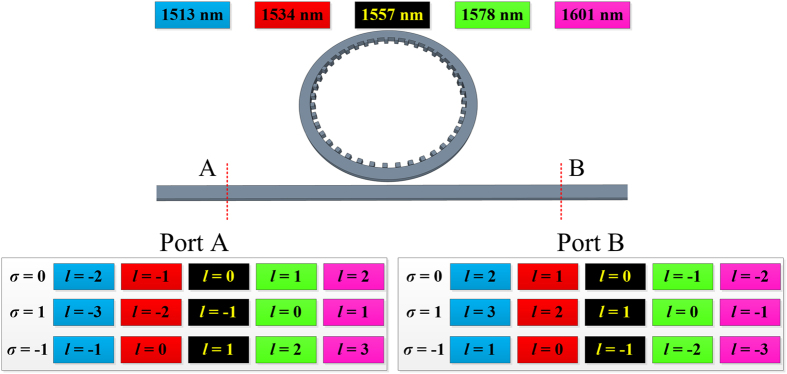
Comparison of resonant wavelengths for vortex beam with different SAMs and TCs based on the detection from Port A and Port B.

It is also interesting to note that the proposed device has the potential to be utilized for multiplexing applications. The results presented above are from the “Port A” detection as depicted in Fig. 4. However, if an opposite cross section located on the other side of the device is used as detection port, the direction of the propagation mode in the ring waveguide will change so will the phase matching condition. The selection rule on “Port B” can be expressed as:


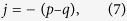


which is also determined by the difference between *p* and *q* but has a minus sign compared with that of “Port A”. Consequently, the resonant wavelength for a specific vortex beam is different at the opposite ports (shown in Fig. 4). This dual-port detection scheme enables the multiplexing of more wavelengths with different OAMs, also has the potential to remove ambiguity that may arise from the single port scheme.

### Reconfigurability of the OAM detector

**Figure 5:**
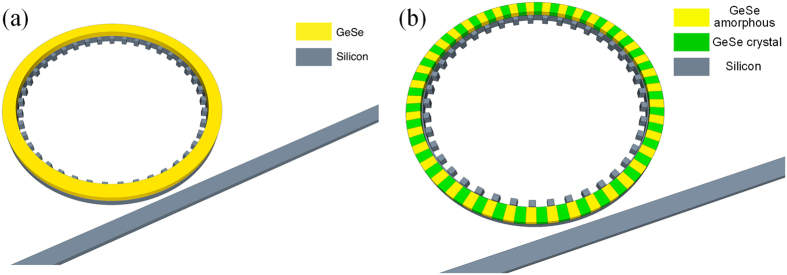
Diagram of the composite OAM receiver that has a (**a**) GeSe annular film and (**b**) GeSe gratings consist of alternate states on top of the resonator as the cladding layer. The duty cycle of the gratings is 0.5.

**Figure 6:**
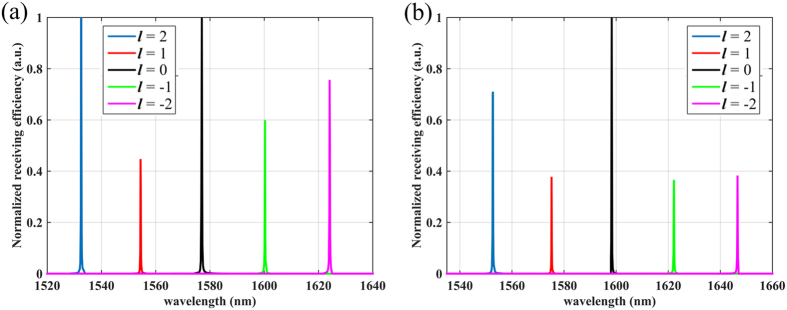
Receiving spectrum of the composited device shown in Fig. 5(a) when the GeSe is in (**a**) amorphous state and (**b**) crystal state, respectively.

As one of the important figure of merit for the critical component in PICs, reconfigurability can improve not only the security but also the adaptability of detectors. As shown in Fig. 2, the differences between adjacent resonances are nearly constant and measured to be about 25 nm. If the peaks can be blue or red shift by an integral multiple of 25 nm, the resonant wavelengths remain unchanged but the related OAM states varies accordingly. In other words, the cavity resonance needs to be tuned according to equation (2) and (4) in order to adjust the detected OAM. However, a minimal mode index change of 0.05 is required to shift the resonance by 25 nm, which is far beyond the capabilities of the conventional materials, but still within the reach for phase change materials such as GeSe. It is worthy of noting that the tunability of similar OAM emitter has been reported by integrating the silicon bend waveguide with addition heater^15^. However, the refractive index change of the silicon induced by the heating effect is very limited, and the difference of the resonant wavelengths between adjacent OAM states increases with decreasing device size. Therefore, the heating mechanism is not suitable for the miniature device proposed in this work. As one type of phase change material that supports high-speed reversible transition between phases, GeSe has been reported recently to offer very large index change and is well compatible with silicon photonics^16^. The unique properties of GeSe provide an opportunity to realize the reconfiguration function of the proposed OAM receiver. As shown in Fig. 5(a), a very thin, annular ‘photonics cap’ on silicon resonator is formed by cladding a GeSe film with thickness of 60 nm. Through doping the GeSe layer and the lower half of the silicon resonator to provide electrical contact for both surfaces of the cladding layer, GeSe film can be pulsed with voltage for phase transition. The refractive indices of amorphous and crystal GeSe are 2.4 + 6e − 5*i* and 2.97 + 6e − 5*i*, respectively^15^. Figure 6 shows the receiving spectrum with the GeSe film in different phases. The applied voltage leads to the phase change of GeSe, then the shift of resonant peaks of nearly 25 nm, enabling the switch of detected OAM between two different states at nearly the same wavelength. In addition, the upper cladding layer offers the function of cancelling the vertical leakage through destructively interfering the leakage propagation mode in the circular resonator^17^, giving rise to a significantly increased quality factor.

**Figure 7:**
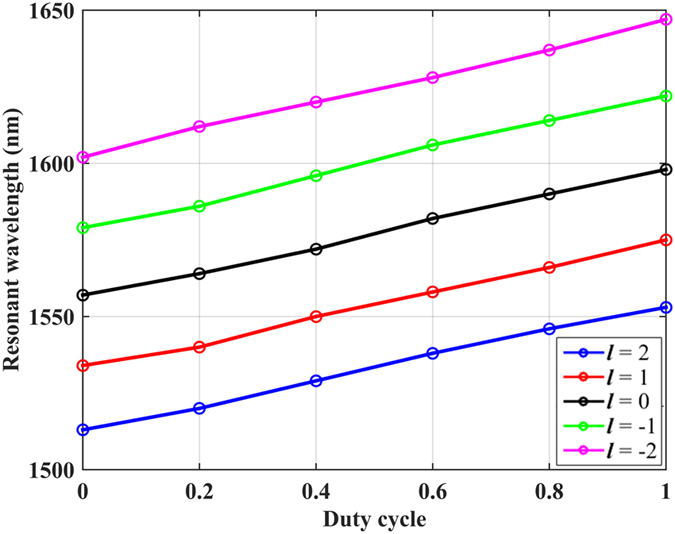
Resonant wavelength of different OAM states versus duty cycle of the GeSe gratings.

Due to the binary phase change nature of GeSe, the modulation range is constant because the mode index can only have two distinct values. More precise control of the resonant wavelength requires the capability to adjust the mode index linearly and continuously within a prescribed range. A feasible solution is to replace the cladding film with gratings, which are consisted of GeSe with alternate states. By patterning dense electrons on the GeSe film, the duty cycle of the gratings can be controlled via turning on different number of electrons. Therefore, the mode index can be adjusted quasi-linearly with the duty cycle according to the effective medium theory. In this case the duty cycle is defined as the ratio between the width of GeSe film in crystal state to the period of the gratings. As an example, 60 nm thick GeSe gratings with duty cycle of 0.5 is deposited on top of the circular resonator and the total number of GeSe grating is chosen to be 41 (shown in Fig. 5(b)). By increasing/decreasing the duty cycle, the area of GeSe film in crystal state increases/decreases as well as the effective mode index, leading to a quasi-continuous shift of the resonant wavelength. The modulation range of the resonant wavelength for specific OAM is determined by the previous results shown in Figs 2 and 4, corresponding to the gratings duty cycle of 0 and 1, respectively. Figure 7 shows the relationship between the resonant wavelength and duty cycle of the GeSe gratings changing from 0 to 1 with increment of 0.2. It can be seen that the resonant wavelength varies quasi-linearly with respect to the duty cycle for arbitrary OAM state. Therefore, this technique enables a quasi-continuous shift of the resonant peak with the sensitivity of nearly 4 nm per 0.1 duty cycle.

## Discussion

In conclusions, we proposed an OAM receiver based on complementary metal oxide semiconductor compatible silicon PICs. This simple and miniature device is capable of receiving distinct and variable OAM states. Besides, its functionality can be easily extended to multiplexing with the dual-port detection technique. The large index change of GeSe enables the reconfiguration of the device, which is realized by adjusting the parameters of top-cladding gratings that consists of GeSe with alternate states. The quasi-linear dependence between the duty cycle of the gratings and the resonant wavelength of the OAM state provides the possibility of modulating the resonance continuously. Such scalable waveguide based OAM receiver is well suitable to be combined with another same device working in the emitting mode so as to build OAM quantum communications channels. This device also provides an approach for an OAM detection architecture that may find important applications in optical communications and information processing with OAM states.

## Additional Information

**How to cite this article**: Rui, G. *et al.* Detection of orbital angular momentum using a photonic integrated circuit. *Sci. Rep.* **6**, 28262; doi: 10.1038/srep28262 (2016).
